# Chitosan/Poly(maleic acid-*alt*-vinyl acetate) Hydrogel Beads for the Removal of Cu^2+^ from Aqueous Solution

**DOI:** 10.3390/gels10080500

**Published:** 2024-07-28

**Authors:** Irina Popescu, Irina Mihaela Pelin, Dana Mihaela Suflet, Magdalena Cristina Stanciu, Marieta Constantin

**Affiliations:** “Petru Poni” Institute of Macromolecular Chemistry, Grigore Ghica Voda Alley 41A, 700487 Iasi, Romania; impelin@icmpp.ro (I.M.P.); dsuflet@icmpp.ro (D.M.S.); cstanciu@icmpp.ro (M.C.S.); marieta@icmpp.ro (M.C.)

**Keywords:** maleic acid copolymer, chitosan beads, copper removal, water treatment

## Abstract

Covalent cross-linked hydrogels based on chitosan and poly(maleic acid-*alt*-vinyl acetate) were prepared as spherical beads. The structural modifications of the beads during the preparation steps (dropping in liquid nitrogen and lyophilization, thermal treatment, washing with water, and treatment with NaOH) were monitored by FT-IR spectroscopy. The hydrogel beads have a porous inner structure, as shown by SEM microscopy; moreover, they are stable in acidic and basic pH due to the covalent crosslinking. The swelling degree is strongly influenced by the pH since the beads possess ionizable amine and carboxylic groups. The binding capacity for Cu^2+^ ions was examined in batch mode as a function of sorbent composition, pH, contact time, and the initial concentration of Cu^2+^. The kinetic data were well-fitted with the pseudo-second-order kinetic, while the sorption equilibrium data were better fitted with Langmuir and Sips isotherms. The maximum equilibrium sorption capacity was higher for the beads obtained with a 3:1 molar ratio between the maleic copolymer and chitosan (142.4 mg Cu^2+^ g^−1^), compared with the beads obtained using a 1:1 molar ratio (103.7 mg Cu^2+^ g^−1^). The beads show a high degree of reusability since no notable decrease in the sorption capacity was observed after five consecutive sorption/desorption cycles.

## 1. Introduction

Heavy metal ions are one of the pollutants that can cause serious environmental problems. Copper has been identified as a major heavy metal contaminant of waste waters because it is a by-product of mining, is widely used in industry (electroplating, paints, pigments, fuel, catalysts, batteries), and in agriculture (in fertilizer, pesticides) [1,2]. In high amounts, copper is toxic to plants, animals, and humans [2]. Like other heavy metal ions, it can be removed from wastewater by different techniques (ion exchange, membrane filtration, etc.) [1], with absorption being one of the most effective methods. Good sorbents should be cost-effective, environmentally friendly, easily regenerated, and have high adsorption capacity and fast kinetics [3,4]. Porous polymeric hydrogels can fulfill these criteria, so they are proposed for these applications [5,6,7]. Compared with larger hydrogels, spherical beads have a higher surface area, and better mass transfer and diffusion behavior. Compared with nano-size sorbents, the beads have the advantages of easy recovery and reusability, properties that are required in water treatment [8].

Chitosan (CS), a cationic polysaccharide derived from chitin, is well-known for its applications in heavy metal ion removal [3,9,10]. CS has the advantages of its abundant availability, low cost, biocompatibility, biodegradability, and, most importantly, its high metal ion adsorption capacity due to the presence of amino and hydroxyl groups. CS’ disadvantages, like solubility in acidic environments and low mechanical properties, can be overcome by physical and chemical modifications [6,9]. Chemical modifications of CS suppose the stabilization of the polymeric network by cross-linking and the addition of new functionalities to increase the sorption capacities [9,11]. Among other derivatizations, grafting carboxylic groups onto CS was used to enlarge the pH solubility domain and increase its sorption properties [9,12]. For example, the polymerization of acrylic acid in the presence of CS and cross-linkers [13,14,15,16] or the grafting of maleic acid on CS [17,18] was used to obtain materials with a high absorption capacity for metal ions.

Copolymers of maleic anhydride with different comonomers (styrene, methyl vinyl ether, acrylic acid, etc.) are known as anti-scale agents, as phosphate substituents in detergents, or for their pharmaceutical applications [19,20]. In the aqueous environment, the hydrolysis of the anhydride cycle from the maleic copolymers leads to the formation of two adjacent carboxylic groups with different acidity constants [21]. The complexation of the two adjacent carboxylic groups with divalent metal ions [22,23] leads to the utilization of the maleic copolymers as anti-scale agents [24], but also for the absorption of the metal ions from wastewater [25,26,27]. By combining maleic acid copolymers with CS, new materials with high metal ion adsorption capacity can be obtained. In our previous work, different maleic copolymers were used in water purification [28]. Among them, poly(maleic acid-alt-vinyl acetate) (MA-VA) proved to be the optimal polymer for obtaining microspheres with the highest dye adsorption capacity. This is why the hydrophilic copolymer MA-VA is proposed in the present study to prepare hydrogels with applications in metal ion removal.

CS and maleic copolymers form polyelectrolyte complexes in aqueous solutions, but the physical electrostatic interactions between the two weak polyelectrolytes are not stable over the entire pH domain [29,30]. Stable chemical interactions are required for reusable materials applied to metal ion absorption. Thus, hydrogels obtained by grafting poly (acrylamide-co-maleic acid) on chitosan by gamma irradiation were recently obtained and used for the removal of cobalt or europium ions [31,32].

In the present paper, the covalent crosslinking between CS and maleic copolymer is performed by an amidation reaction. The strategy used to prepare stable porous spherical beads was the dropping of the CS/MA-VA mixture solution into liquid nitrogen, followed by lyophilization. Thermal treatment of the dried beads will induce covalent bonds between the polymers. Two ratios between the polymers were used for the preparation of the beads to evaluate how the ratio between the amine groups of CS and the carboxylic groups of the maleic copolymer influences the absorption of Cu^2+^ ions. The structural modifications during the preparation steps were monitored by FT-IR spectroscopy. The morphology of the beads and their swelling behavior were followed. The sorption capacity of the new materials for Cu^2+^ removal was investigated as a function of pH, contact time, and the initial concentration of metal ions. For a better understanding of Cu^2+^ sorption mechanisms, the sorption kinetics and isotherms were fitted with different models. The reusability of the CS/MA-VA beads was also evaluated. To our knowledge, this is the first time CS/MA-VA hydrogels were obtained and employed for metal ion removal.

## 2. Results and Discussion

### 2.1. Preparation and Characterization of CS/MA-VA Beads

**Figure 1:**
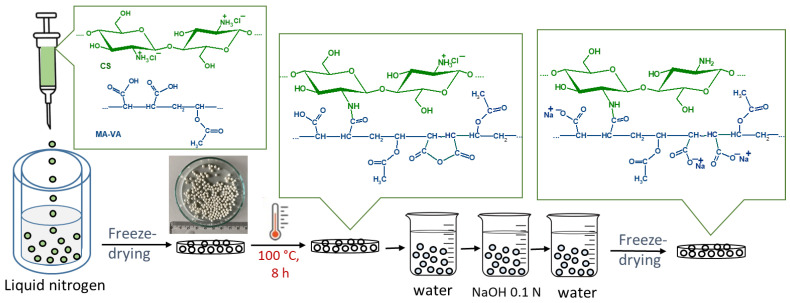
Schematic representation of the preparation process of CS/MA-VA beads.

CS and MA-VA aqueous solutions were mixed, both polymers being in a protonated state to avoid the formation of a polyelectrolyte complex. Then, the polymeric solution was dripped into liquid nitrogen, and the resulting beads were dried by lyophilization. Subsequently, the beads were cross-linked by the amidation reaction between the amine groups of CS and the carboxylic groups of MA-VA copolymers under thermal treatment (Figure 1). The beads were washed with distilled water to remove the uncross-linked polymers, and then with alkaline water to dissociate the remaining carboxylic groups of the maleic copolymer. Following this treatment, the amine groups of CS chains are in a non-protonated state, the optimal state that can be involved in the chelation with metal cations [9].

**Figure 2:**
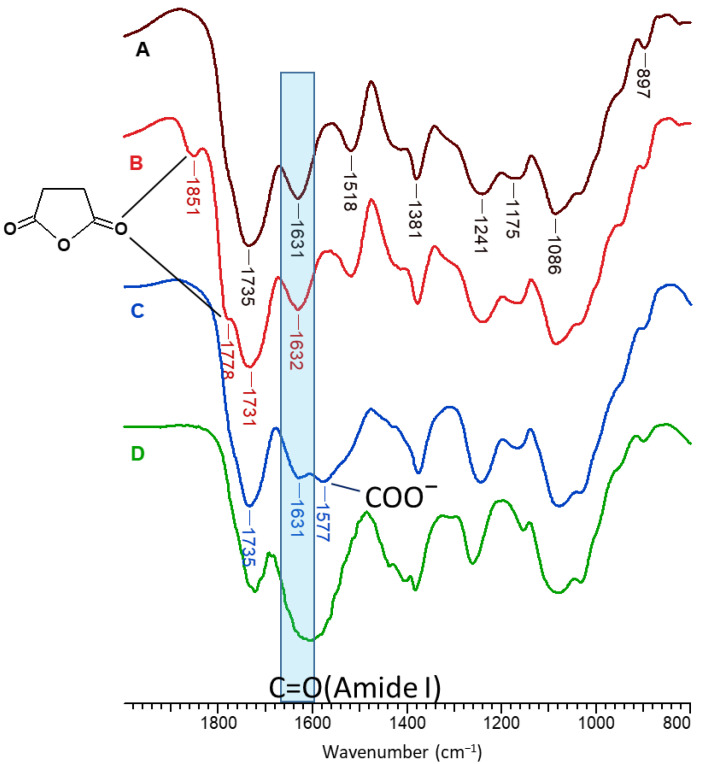
FT-IR spectra in the 2000–800 cm^−1^ region of CS/MA-VA beads after the first freeze-thawing (**A**), after thermal treatment at 100 °C for 8 h (**B**), after washing (**C**), and after the treatment with NaOH and washing with distilled water (**D**).

FT-IR spectroscopy was used to elucidate the structure of the beads after each treatment. Figure 2 presents the FT-IR spectra of the beads after the first freeze-drying (A), thermal treatment (B), anhydride hydrolysis (C), and finally treatment with NaOH (D). In the spectrum of CS/MA-VA beads initially obtained (Figure 2A), the characteristic bands for both CS and MA-VA copolymers can be observed at: 1735 cm^−1^ (carbonyl groups from maleic acid and vinyl acetate units), 1631 cm^−1^ (amide I from CS), 1518 cm^−1^ (–NH_3_^+^ groups from CS chloride), 1381 cm^−1^ (–CH_3_ from vinyl acetate), 1241 and 1175 cm^−1^ (–C–O stretching from vinyl acetate), 1086 and 897 cm^−1^ (C–O vibration from CS) [33,34,35,36]. After the thermal treatment (Figure 2B), new bands appeared at 1851 and 1778 cm^−1^ because of the de-hydration and re-formation of the anhydride cycle in the maleic copolymer [36]. The amide bonds formed between the carboxylic groups of the maleic copolymer and the amine groups in CS are overlapped by the amide bonds in CS. After washing the beads with distilled water (Figure 2C), the anhydride cycles are hydrolyzed with the obtaining of carboxylic groups, and the bands from 1851 and 1778 cm^−1^ were not observed anymore. The appearance of a new band at 1577 cm^−1^ can be due to the ionization of some of the carboxylic acid groups. After treatment with NaOH and washing with distilled water (Figure 2D), a large band can be observed between 1680 and 1480 cm^−1^ attributed to the amide bonds, dissociated –COO^−^ groups from the maleic copolymer, and also –NH_2_ groups from CS.

Two molar ratios between the CS and maleic copolymer were used to prepare the beads, as shown in Table 1. In order to determine the cross-linking degree of CS, the ninhydrin test was used [37]. The content of free –NH_2_ groups in CS was determined before and after thermal treatment (Table 1). The difference between these two values is given by the amine groups involved in the amidation reactions that take place under the thermal treatment.

The gel fractions were relatively high for both samples, showing that the bead structure is stabilized not only by ionic interaction but also by the covalent cross-linking between the CS and MA-VA copolymers.

**Figure 3:**
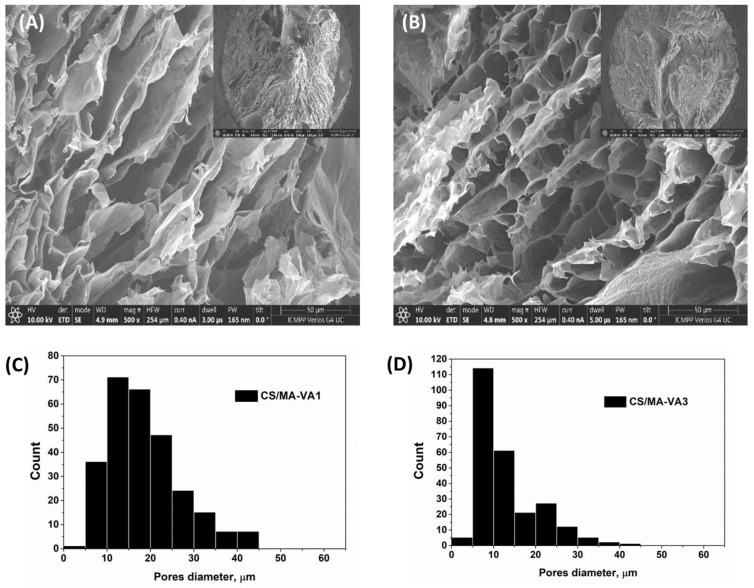
SEM images (cross-sections) of CS/MA-VA1 (**A**) and CS/MA-VA3 beads (**B**) with high and low (inset images) magnification, together with pore size distribution diagrams (**C**,**D**).

The beads have spherical shapes, with diameters in their dried state ranging between 2.4 and 3.8 mm. SEM images (Figure 3) showed that both samples have a porous inner structure with lamellar and interconnected pores, characteristic of beads prepared in liquid nitrogen and dried by lyophilization [38]. Compared with CS/MA-VA1 beads with a mean pores diameter of 20.1 µm, the CS/MA-VA3 beads have smaller pores (mean diameter 12.6 µm) and a narrower pores distribution (Figure 3C,D). This is due to the higher cross-linking degree of CS/MA-VA3 beads through amide linkage and electrostatic interaction.

**Figure 4:**
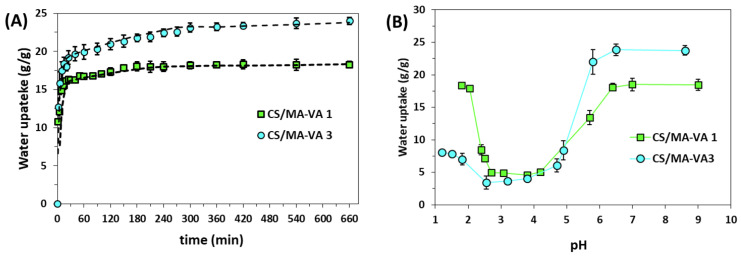
Swelling kinetics of CS/MA-VA beads in pure water (**A**) and the influence of the final pH on the swelling capacity at equilibrium (**B**).

It is widely recognized that a high swelling capacity is essential for a good sorbent [5]. However, a too-high swelling degree can make materials brittle and fragile [39,40]. This drawback can be overcome by an increase in the cross-linking density, which lowers the hydrophilicity of the polymer [39]. Cross-linking can enhance the resistance of the polymer against acid, alkali, and chemicals but reduce the swelling degree and the efficiency of the uptake of pollutants. Therefore, the swelling kinetics of CS/MA-VA beads were performed. As shown in Figure 4A, both CS-MA-VA1 and CS-MA-VA3 beads absorb high amounts of water in the first few minutes, then gradually swell and reach equilibrium in about 6 h. However, the CS/MA-VA3 beads, even if they show a high degree of cross-linking, have the highest swelling ratio due to the large content of dissociated carboxylic groups at the pH value of distilled water (5.4).

The influence of the pH on the swelling capacity at equilibrium was studied for CS/MA-VA1 beads and presented in Figure 4B. It is known that the intrinsic dissociation constants for MA-VA copolymers in pure water are pK_a_^1^ = 4.2, and pK_a_^2^ = 7.3. Nevertheless, added salts or polycations can force the dissociation of the carboxylic groups, decreasing the pK_a_ values (i.e., pK_a_^1^ = 3.4, pK_a_^2^ = 5.6 in the presence of 0.1 M LiCl) [20]. The protonation constant for CS is approximately 6.5 [41].

At a pH of around 2, the carboxylic groups of the maleic copolymer are not dissociated, but the amine groups from CS are protonated, which determines the swelling of the beads (Figure 4B). In this form, the beads with a higher content of CS (CS/MA-VA1) have the highest swelling degree. When the pH is between 3 and 4, half of the carboxylic groups and the amine groups of CS are ionized and interact with each other, leading to the collapsing of the beads (polyelectrolyte complexation). When the pH increases above 4.2, more and more carboxylic groups begin to dissociate. A higher number of dissociated carboxylic groups compared with the ionized –NH_3_^+^ groups of CS determines the swelling of the beads. Even if the amine groups of CS are in the –NH_2_ form at pH over 6.4, the high swelling of the beads is assured by the carboxylic groups of the maleic copolymer that are all dissociated at basic pH. In this form, the CS/MA-VA3 beads with the highest content of carboxylic groups show the highest swelling capacity.

### 2.2. Cu^2+^ Sorption

#### 2.2.1. Influence of Initial pH

**Figure 5:**
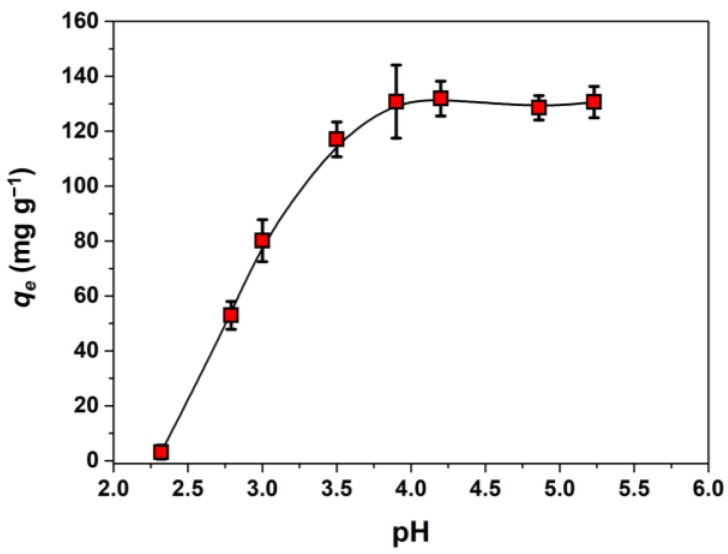
Influence of the initial pH on the sorption capacity of CS/MA-VA3 beads for Cu^2+^ ions. (*C*_0_ = 320 mg L^−1^, sorbent dose 1 g L^−1^, contact time 24 h).

The pH value of solutions affects the charge of the adsorbent and the speciation of heavy metal ions, thus being one of the factors that influence sorption. The adsorption of Cu^2+^ ions by the CS/MA-VA3 beads was studied in the acidic pH range of 2–5.2 (Figure 5). The initial pH of Cu^2+^ solution could not be further increased due to the precipitation of the formed Cu(OH)_2_. As seen from Figure 5, the sorption capacity increases with the increase of the initial pH from 2.3 to 4, and remains constant at a pH between 4 and 5.2. Similar results could be seen in the literature for sorbents based on CS [42,43] or CS and anionic polymers containing carboxylic groups [14,31,44]. The complexation between Cu^2+^ and CS was shown to take place at pH > 4 [45] or even at pH > 5.3 [46] when CS has non-protonated amine groups that are involved in the chelation with metal cation through the free electron doublet of nitrogen [9]. In addition, the increase in pH leads to the ionization of the polymeric carboxylic groups that will electrostatically attract the Cu^2+^ cations from the solution.

#### 2.2.2. Sorption Kinetics

**Figure 6:**
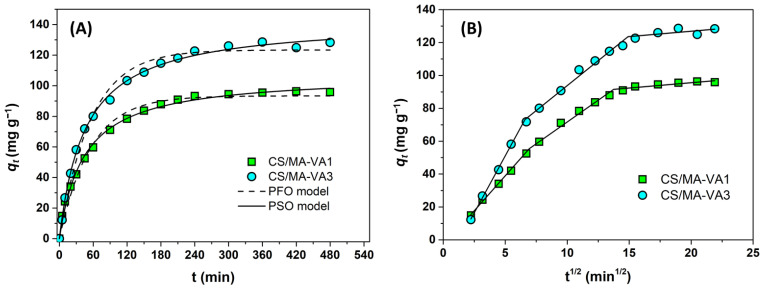
Sorption kinetics and nonlinear fitting of Cu^2+^ ions onto CS/MA-VA beads (**A**), together with the Weber and Morris intra-particle diffusion representation (**B**) (*C*_0_ = 320 mg L^−1^, initial pH 4.7, sorbent dose 1 g L^−1^, 25 °C).

Figure 6A presents the effect of contact time on the Cu^2+^ retention and shows that the sorption equilibrium is achieved after 6 h.

To elucidate the sorption kinetics, the experimental data were fitted by pseudo-first-order (Equation (1)), pseudo-second-order kinetics (Equation (2)), and the intra-particle diffusion Weber and Morris model (Equation (3)). These models are based on the following equations [47,48]:

(1) $$q_{t}=q_{e}\left(1-e^{-k_{1}t}\right)$$

(2) $$q_{t}=k_{2}q_{e}^{2}t/\left(1+k_{2}q_{e}t\right)\;$$

(3) $$q_{t}=k_{dif}t^{1/2}+A$$

where $q_{t}$ (mg g^−1^) and $q_{e}$ (mg g^−1^) are the masses of Cu^2+^ adsorbed per gram of beads at time t and at equilibrium, respectively. $k_{1}$ (min^−1^), $k_{2}$ (g mg^−1^ min^−1^), and $k_{dif}$ (g mg^−1^ min^−0.5^) represent the rate constants of pseudo-first-order, pseudo-second-order and intra-particle diffusion models, respectively. The constant *A* (mg g^−1^) is related to the diffusion resistance.

The pseudo-first-order (PFO) model is applied at a high initial concentration of sorbate (*C*_0_) and when the adsorption is controlled by external and internal diffusion. The pseudo-second-order (PSO) model is applied at low *C*_0_ and when the sorption is controlled by adsorption on the abundant active sites [41]. The non-linear fitting of the experimental data with the PFO and PSO models is shown in Figure 6A, and the obtained kinetic parameters and the correlation coefficients (R^2^) are presented in Table 2. The higher values of R^2^ show that the PSO model described the Cu^2+^ sorption onto CS/MA-VA beads better than the PFO model. This means that the rate-control mechanism is chemisorption, in agreement with other reports of metal ion adsorption on chitosan-based materials with or without carboxylic groups [13,16,42,43,49,50]. In the case of CS/MA-VA beads, the chemisorption may be accomplished by chelating Cu^2+^ ions with the –COO^−^, –NH_2_, and –OH groups. The $q_{e}$ values calculated with the PSO model agree with the experimental values (Table 2). From the *k*_2_ and *q_e_* values, it can be concluded that the adsorption is faster for the CS/MA-VA1 sample, but the adsorption capacity is higher for the beads containing a high amount of carboxylic groups (CS/MA-VA3).

The intra-particle diffusion model, proposed by Weber and Morris, was further applied to identify the diffusion mechanism during the sorption process. Figure 6B shows the representation of the Cu^2+^ adsorption amount versus the square root of time ($q_{t}$ vs. $t^{1/2}$). It is known that if this dependence is linear and passes through the origin, the intra-particle diffusion is the controlling process; otherwise, the adsorption is controlled by multiple processes [47,51]. For Cu^2+^ sorption onto CS/MA-VA beads, three different slopes are required to fit the experimental data, indicating that the process involves three stages. Generally, the last process (with the lowest slope) is attributed to the equilibrium phase, where the low concentration of sorbate in solution and the fewer adsorption sites determine the slowing down of intraparticle diffusion [51,52,53]. The first two stages can be attributed to (1) external surface adsorption, (2) intraparticle diffusion [42,51,52,53,54], or (1) macropore diffusion, and (2) micropore diffusion [55,56]. Taking into account the high porosity of CS/MA-VA beads, demonstrated by SEM, and the fact that the initial adsorption phase takes place in the first 40 min when a high swelling rate of the beads was observed (Figure 4A), the explanation involving macropores and micropore diffusion seems more plausible for the sorption of Cu^2+^ ions from aqueous solution in the first two stages. The values of rate parameters for the three stages are given in Table 2, with *k_dif_*_,1_ > *k_dif_*_,2_ > *k_dif_*_,3_.

#### 2.2.3. Sorption Isotherms

**Figure 7:**
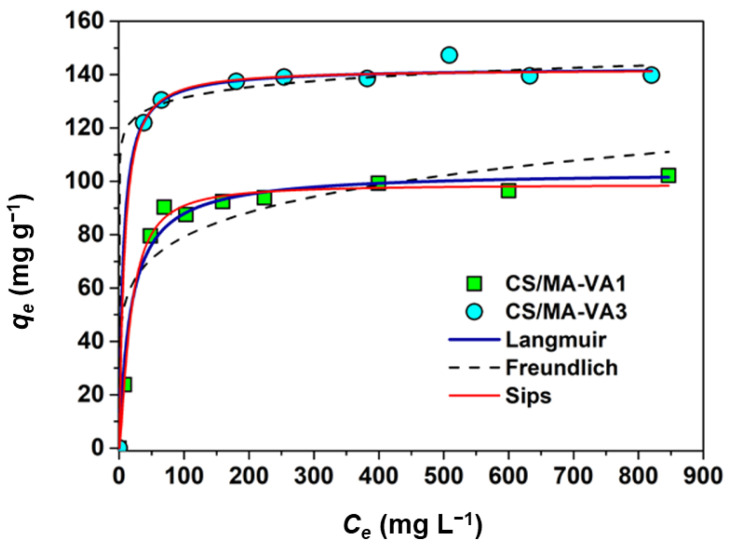
Equilibrium sorption isotherms for the Cu^2+^ sorption onto CS/MA-VA beads (initial pH = 4.7, sorbent dose 1 g L^−1^, 25 °C).

The sorption at equilibrium as a function of Cu^2+^ concentration at equilibrium was presented in Figure 7. The experimental data were fitted with three isotherm models: Langmuir (Equation (4)), Freundlich (Equation (5)), and Sip (Equation (6)) [4].

(4) $$q_{e}=\frac{q_{max}K_{L}C_{e}}{1+K_{L}C_{e}}$$

(5) $$q_{e}=K_{F}C_{e}^{1/n}$$

(6) $$q_{e}=\frac{q_{max}K_{S}C_{e}^{1/n}}{1+K_{S}C_{e}^{1/n}}$$

where $q_{e}$ (mg g^−1^) is the Cu^2+^ sorption at equilibrium, $C_{e}$ (mg L^−1^) is the concentration of the solute at equilibrium in liquid phase, $q_{max}$ (mg g^−1^) is the maximum adsorption capacity, $K_{L}$ (L mg^−1^) is the Langmuir equilibrium constant,$\;K_{F}$ ((mg g^−1^)(L mg^−1^)^1/n^) is the Freundlich constant, $K_{S}$ (L mg^−1^)^1/n^) is the Sips constant, and *n* (dimensionless) is a constant in Freundlich and Sips models regarded as a measure of the system heterogeneity.

It is known that the Langmuir model (based on the assumption that monolayer adsorption occurs on the homogeneous surface with energetically equivalent adsorption sites) is characterized by a plateau at high sorbent concentrations, while the Freundlich model (assuming multilayer adsorption on the energetic heterogeneous adsorption surface) better fits the experimental data in the moderate concentration range, while the Sips model combines the Langmuir and Freundlich models [4].

The non-linear fitting of the experimental data with these models (Figure 7) led to the parameters presented in Table 3. The high values of R^2^ show that Langmuir and Sips isotherms describe very well the Cu^2+^ sorption onto CS/MA-VA beads. The *K_L_* and *K_S_* values are higher for CS/MA-VA3 beads compared with CS/MA-VA1 beads, showing that the adsorption of Cu^2+^ is more favorable for polymeric materials possessing a higher amount of carboxylic groups. This can also be seen from the shape of the isotherms: for CS/MA-VA1 beads, the isotherm has an L-shape (Langmuir type) characteristic of favorable adsorption, and for CS/MA-VA3 beads, the shape of the isotherm is more like the H-shape (high affinity) characteristic of strongly favorable sorption [4,57]. The Langmuir isotherm model predicts a maximum adsorption capacity (*q_max_*) of 103.7 mg Cu^2+^ g^−1^ and 142.4 mg Cu^2+^ g^−1^ for CS/MA-VA1 and CS/MA-VA3, respectively. The maximum adsorption capacities for Cu^2+^ found in the literature for different materials based on maleic acid copolymers, or CS, are presented in Table 4. From the analysis of these data, we can conclude that the *q_max_* value obtained for CS/MA-VA3 beads is generally higher than those obtained for the materials based on maleic acid copolymers [25,26,27,58,59,60] or native CS [50,61,62,63,64,65], and comparable with the materials containing CS together with other chelating groups (phosphate, carboxyl, L-arginine, amidoxime, xanthate, etc.) [15,16,17,18,49,53,66,67,68,69,70,71,72].

#### 2.2.4. Adsorption Thermodynamics

The values of the enthalpy change (Δ*H*), entropy change (Δ*S*), and Gibbs free energy (Δ*G*) were calculated using the equations:

(7) $$lnK_{d}=\frac{∆S}{R}-\frac{∆H}{R\cdot T}$$

(8) $$∆G=-R\cdot T\cdot lnK_{d}$$

where $K_{d}$ is the distribution constant at equilibrium ($K_{d}=q_{e}/c_{e})$, T is the temperature in Kelvin, and R is the ideal gas constant (8.314 J K^−1^ mol^−1^).

Experiments were carried out for CS/MA-VA beads at 298, 308, and 318 K, and the thermodynamic parameters obtained by representing $lnK_{d}$ as a function of 1/*T* are given in Table 5.

The negative values of Δ*G* illustrate spontaneous and favorable sorption at these temperatures. The sorption process is endothermic (Δ*H* > 0), meaning that temperature increases are favorable for the adsorption of Cu^2+^ onto CS/MA-VA beads. This finding agrees with other thermodynamic studies of copper sorption onto CS-based hydrogels [16,72].

#### 2.2.5. Characterization of the Beads after Sorption

**Figure 8:**
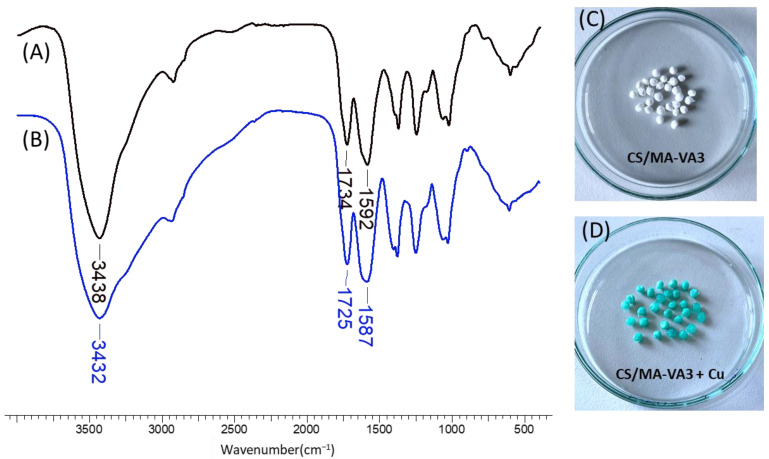
FT-IR spectra of CS/MA-VA3 beads before (**A**) and after Cu^2+^ sorption (**B**), together with their optical images (**C**,**D**).

The first indication of Cu^2+^ sorption onto CS/MA-VA beads is the appearance of the blue color, as shown in Figure 8. The mechanism of Cu sorption was studied by FT-IR spectroscopy. As shown in Figure 8, there are some differences in the spectra of CS/MA-VA3 beads before and after Cu^2+^ loading. The broad adsorption band from 3438 cm^−1^ attributed to the O-H and N-H bonds moved to 3432 cm^−1^ and broadened after Cu^2+^ sorption, suggesting that the hydroxyl and amine groups from CS are involved in the interaction with the metal ion [15,17,73]. The adsorption bands at 1592 cm^−1^ and 1734 cm^−1^ assigned to the carbonyl from the dissociated –COO^−^ and acidic –COOH groups, respectively, are shifted to lower wavenumbers (1587 and 1725 cm^−1^, respectively) in the spectrum of the beads loaded with Cu^2+^. This fact proved that the carboxylic groups from the maleic acid copolymer are also involved in the electrostatic interaction and coordination with the divalent metal [17,73,74].

The compression tests showed that the mechanical properties of the beads were modified after Cu^2+^ loading. The forces required to break the unloaded wet spherical beads were 1.25 ± 0.08 N and 1.72 ± 0.18 N for the CS/MA-VA1 and CS/MA-VA3 beads, respectively. In contrast, the beads after Cu^2+^ sorption can be compressed with 40 N without breaking but only undergoing plastic deformation, showing that the chelation acts as a further crosslinking agent and increases the mechanical properties of the beads.

**Figure 9:**
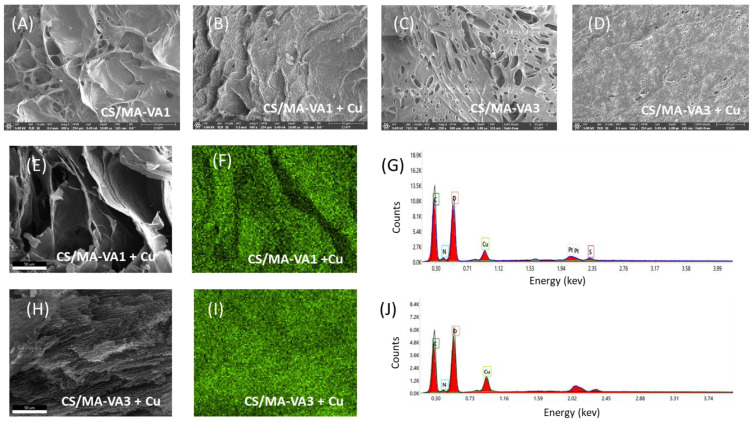
SEM images of the surface of the CS/MA-VA1 (**A**,**B**) and CS/MA-VA3 beads (**C,D**) before (**A**,**C**) and after copper sorption (**B**,**D**). SEM images of the copper-loaded beads in cross-section (**E**,**H**) together with the elemental mapping of Cu (**F**,**I**) and EDX profiles in the section of the CS/MA-VA1 (**G**) and CS/MA-VA3 beads (**J**).

The morphology of the beads surface and cross-section before and after copper sorption was studied by SEM. The surface of the CS/MA-VA beads was modified after the adsorption of Cu^2+^ (Figure 9A–D). Due to the mass transfer of copper ions onto the beads, the surface appeared denser and smoother, and a reduction of the pores was observed.

The adsorption of Cu^2+^ in the volume of CS/MA-VA beads was confirmed by energy-dispersive X-ray analysis (EDX) in cross-section (Figure 9E–J). The EDX elemental images of Cu presented in Figure 9F,I show that the metal was absorbed in the porous structure of the beads and was uniformly distributed. The mass percent of copper was 8.4 ± 0.9% and 12.3 ± 1.6% in the loaded CS/MA-VA1 and CS/MA-VA3 beads, respectively. Sulfur from Cu(SO_4_) was also present in low amounts in the beads after sorption.

#### 2.2.6. Reusability

**Figure 10:**
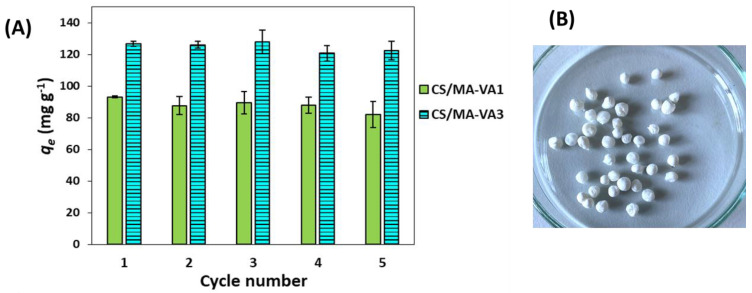
Influence of the sorption/desorption cycle on the sorption performance of CS/MA-VA beads (initial Cu^2+^ concentration 320 mg L^−1^, initial pH = 4.7, sorbent dose 1 g L^−1^, 25 °C) (**A**). Optical image of the CS/MA-VA3 beads after 5 sorption/desorption steps (**B**).

The reusability of the materials used for the sorption of metal ions from aqueous solutions is a crucial factor from a practical and economical point of view. Therefore, in the present study, the desorption of Cu^2+^ ions from the CS/MA-VA beads was performed with 0.1 M HCl, and the beads were regenerated with 0.1 M NaOH to re-dissociate the carboxylic groups and deprotonate the amine groups. After regeneration, the beads were used for another sorption cycle, and the equilibrium sorption capacity during consecutive sorption/desorption cycles was determined. As Figure 10A shows, the adsorption of Cu^2+^ ions at equilibrium remained almost unchanged after the fifth cycle of sorption, both for CS/MA-VA1 and CS/MA-VA3.

The chemical crosslinking between the CS and MA-VA copolymers allows the beads to be exposed to different pHs while maintaining their integrity after five sorption/desorption cycles. After desorption, the beads lose the blue color given by copper (Figure 10B).

## 3. Conclusions

Hydrogel beads based on CS and MA-VA copolymers were obtained as new sorbent materials that cumulate the properties of both natural polycation and synthetic polyanion. The obtained beads are porous, with pores sizes ranging between 5 and 40 µm, allowing fast swelling and high water uptake values. The polymeric network of the beads is stable at acidic and basic pHs due to the covalent crosslinking between the amine and carboxylic groups.

The sorption of Cu^2+^ from aqueous solution onto CS/MA-VA beads is higher at an initial pH between 4 and 5.2. The sorption kinetics data are better fitted with the PSO model, meaning that the rate-control mechanism is chemisorption. The sorption equilibrium data were better fitted by Langmuir and Sips isotherms. The maximum equilibrium sorption capacity was higher for the CS/MA-VA3 beads possessing higher amounts of carboxylic groups (142.4 mg Cu^2+^ g^−1^), compared with the CS/MA-VA1 beads with a higher amount of amine groups (103.7 mg Cu^2+^ g^−1^). This difference demonstrates that interaction between carboxylic groups and the metal cations brings a greater contribution than that given by the coordination between -NH_2_ and –OH groups from CS and the metal ions.

Desorption of Cu^2+^ from the CS/MA-VA beads was easily performed with 0.1 M HCl, and then the adsorptive sites were regenerated with 0.1 M NaOH. The hydrogel beads kept their sorption capacity and physical integrity after five sorption/desorption cycles, proving their reusability. In conclusion, the obtained hydrogel beads with good adsorption capacity and reusability can be regarded as new sorbents for Cu^2+^ retention from wastewater.

## 4. Materials and Methods

### 4.1. Materials

Chitosan (CS) of low molecular weight was purchased from Sigma-Aldrich Chemie GmbH (Steinheim, Germany). The degree of de-acetylation, determined by NMR, was 80.18%, and the molecular mass was 240 kDa, as determined by viscometric measurements [75]. Poly(maleic anhydride-alt-vinyl acetate) was obtained by radical polymerization [76], and the co-monomer molar ratio in the copolymer was 1:1, as determined by conductometric titration in acetone:water (1:1, *v*/*v*) [20]. The molecular mass of the copolymer determined by viscometric measurements [77] was found to be 99 kDa.

CuSO_4_ · 5H_2_O was purchased from Chemical Company (Iasi, Romania). Polyethylene imine (PEI) solution (50%, *w*/*v*), ninhydrin, hydrindantin, and lithium acetate were purchased from Sigma-Aldrich (Steinheim, Germany). Distilled water was used in all the experiments.

### 4.2. Beads Preparation

A 3 wt. % CS hydrochloride solution was prepared as follows: CS with 4.73 meq NH_2_ g^−1^ was dispersed in water, a calculated volume of 1 N HCl was added (to dissociate the amine groups), and the CS hydrochloride solution was stirred overnight. A 10 wt. % MA-VA aqueous solution was obtained by solving poly(maleic anhydride-*alt*-vinyl acetate) in water overnight when the hydrolysis of the anhydride cycles took place. The two solutions were mixed in different ratios, and the mixture was dropped into liquid nitrogen through a 21 G metal needle at a 30 mL h^−1^ flow rate. The obtained cryo-beads were lyophilized using an ALPHA 1–2LD freeze-drier (Martin Christ, Germany). The covalent cross-linking between the NH_2_ groups of CS and –COOH groups of maleic acid copolymer was performed by keeping the beads for 8 h at 100 °C and reducing the pressure (0.1 atm) to remove by-product water. The hydrogel beads are sequentially washed with distilled water to remove the un-crosslinked polymers, diluted NaOH solution (to deprotonate the amine groups of CS and dissociate the carboxylic groups of the maleic copolymer), washed again with distilled water, and dried by lyophilization. Two different bead samples were prepared: CS/MA-VA1 and CS/MA-VA3, obtained with a 1:1 and 1:3 molar ratio between the amine groups of CS and maleic units from MA-VA, respectively (Table 1).

### 4.3. Beads Characterization

FT-IR spectra of the beads were recorded on an FT-IR Vertex 70 spectrometer (Bruker, Austria) in KBr. Scanning Electron Microscopy (SEM) was performed using a Verios G4 UC scanning electron microscope (Thermo Fisher Scientific, Brno, Czech Republic) with an ETD detector, and for the energy-dispersive X-ray analysis (EDX), the Octane Elect Super SDD detector was used. Before the SEM analysis, the samples were coated with platinum using a Leica EM ACE200 Sputter coater (Leica Microsystems, Vienna, Austria) to prevent charge buildup during exposure to the electron beam.

The gel fraction (*GF*) was calculated using the weight of the dried beads after the thermal treatment (*w*_0_) and the weight of the dried beads after washing (*w_w_*):

(9) $$GF=\frac{w_{w}}{w_{0}}\times 100$$

The ninhydrin assay was performed to determine the content of the free primary amino groups of CS that were not involved in the cross-linking reaction after the thermal treatment [78,79]. The ninhydrin reagent solution was prepared according to the literature [66] using ninhydrin and hydrindantin solved in DMSO and mixed with lithium acetate buffer. Over the beads (5 mg) swollen in 5% acetic acid (5 mL), ninhydrin reagent (5 mL) was added. The mixture was boiled in water for 30 min, when an intense blue coloration appeared. After dilution with ethanol: water (50:50, v:v), the absorbance at 570 nm was measured using an Evolution 201 UV-visible spectrophotometer (Thermo Scientific, Waltham, MA, USA). The content of amine groups was obtained using a previously obtained calibration curve using glycine as a standard.

To determine the swelling kinetics, the dried beads (0.1 g) were soaked in water. At different time intervals, the beads were withdrawn and weighed after removing the superficial water with filter paper. The water uptake (W) was calculated as:

(10) $$W=\frac{w_{s}-w_{d}}{w_{d}}$$

where $w_{d}$ and $w_{s}$ are the weights of the dried and swollen beads, respectively, measured at different times.

When the influence of the pH on the swelling was studied, the beads were soaked in water, and the pH was modified with 0.1 N NaOH or 0.1 N HCl solutions. After 24 h, the swelled beads were weighed, and the pH of the solution was measured again. Water uptake is represented as a function of the final pH.

Uniaxial compression of single wet beads was carried out using a texture analyzer (Brookfield Texture PRO CT3^®^, Brookfield Engineering Laboratories Inc., Middleboro, MA, USA) with a 50 N force transducer. The compression was performed with up to 80% deformation at a speed of 0.5 mm/s. 15 beads from each sample were compressed in order to give statistically representative results.

### 4.4. Cu^2+^ Sorption

Metal ion retention studies were estimated using the batch technique. About 0.05 g of dried beads were placed in flasks containing 50 mL of a solution of 320 mg Cu^2+^ L^−1^ (5 mM CuSO_4_). The flasks were shaken at 120 rpm at room temperature for 24 h for the equilibrium studies and for different time intervals in the case of the kinetic studies. The experimental adsorption isotherms were obtained by varying the initial concentration of Cu^2+^ between 30 mg Cu^2+^ L^−1^ and 950 mg Cu^2+^ L^−1^ at pH 4.7 and at room temperature (25 °C).

The concentration of Cu^2+^ was determined colorimetrically with PEI using the absorbance of the PEI-Cu^2+^ complex [80]. Briefly, a PEI solution (1.075 g L^−1^) was first prepared. Then 4 mL of this solution was added into volumetric flasks (10 mL) together with different volumes of CuSO_4_ solution, brought to 10 mL with distilled water, and the UV-visible spectra were recorded. Thus, in the measured solutions, the concentration of PEI was 430 mg L^−1^, and for the calibration curve, Cu^2+^ concentration ranges from 0.5 to 16 mg L^−1^ (y=0.0621·x, where y is the absorbance at 273 nm and x is the concentration of Cu^2+^ in mg L^−1^).

Cu^2+^ sorption capacity at equilibrium ($q_{e}$, mg g^−1^) was calculated as:

(11) $$q_{e}=\frac{\left(C_{0}-C_{e}\right)V}{W_{d}}$$

where $C_{0}$ is the initial metal ion concentration (mg L^−1^), $C_{e}$ is the concentration of the metal ions in the solution at equilibrium (after absorption), *V* is the volume of the aqueous phase, and $W_{d}$ is the weight of dried beads (g).

### 4.5. Regeneration and Reusability

0.05 g beads were incubated with 50 mL Cu^2+^ solution (320 mg L^−1^). After equilibrium (24 h), the beads were withdrawn, washed with 20 mL 0.1 M HCl for 2 h for the desorption of Cu^2+^, washed with distilled water, regenerated with 0.1 M NaOH for 2 h, and washed with distilled water until the neutral pH was reached. The beads were dried by freeze-thawing and re-used for other absorption cycles.

## Figures and Tables

**Table 1:** Preparation conditions and the main characteristics of the resulting beads.

Sample	Initial Reaction Mixture		Beads Characterization		
	NH_2_/Maleic Units Molar Ratio	CS/Maleic Copolymer (wt/wt)	NH_2_ Content (meq g^−1^) Before/After Thermal Treatment	Crosslinking Degree of Amine Groups (%)	Gel Fraction (%)
CS/MA-VA1	1/1	53.4/46.6	0.75 ± 0.02/0.64 ± 0.02	14.6 ± 5.0	81.7 ± 1.5
CS/MA-VA3	1/3	27.7/72.3	0.48 ± 0.01/0.38 ± 0.02	20.8 ± 5.9	99.1 ± 0.4

**Table 2:** Kinetic data for Cu^2+^ sorption on the CS/MA-VA beads.

	CS/MA-VA1	CS/MA-VA3
*q_e,exp_* (mg g^−1^)	96.3	137.5
PFO model		
*q_e,calc_* (mg g^−1^)	93.4	123.4
*k*_1_ (min^−1^)	0.018	0.018
R^2^	0.984	0.987
PSO model		
*q_e,calc_* (mg g^−1^)	105.3	142.0
*k*_2_ (g mg^−1^ min^−1^)	2.13	1.53
R^2^	0.995	0.997
W&M intra-particles diffusion model		
*k_dif_*_,1_ (g mg^−1^ min^−0.5^)	8.5	13.8
*k_dif_*_,2_ (g mg^−1^ min^−0.5^)	5.3	6.1
*k_dif_*_,3_ (g mg^−1^ min^−0.5^)	0.6	0.7

**Table 3:** Isotherm parameters of Langmuir, Freundlich, and Sips models obtained by non-linear fitting for the sorption of Cu^2+^ onto CS/MA-VA beads.

	CS/MA-VA1	CS/MA-VA3
Langmuir model		
*q_max_* (mg g^−1^)	103.7	142.2
*K_L_* (L mg^−1^)	0.056	0.159
R^2^	0.983	0.996
Freundlich model		
*K_L_* ((mg g^−1^)(L mg^−1^)^1/n^)	36.27	108.4
1/*n*	0.158	0.041
R^2^	0.851	0.989
Sips model		
*q_max_* (mg g^−1^)	98.8	141.9
*K_S_* ((L mg^−1^)^1/n^)	0.018	0.101
1/*n*	1.40	1.13
R^2^	0.993	0.996

**Table 4:** Comparison of maximum equilibrium adsorption capacity of Cu^2+^ on different materials based on maleic acid copolymers or/and CS.

Sorbent	*q_max_* (mg Cu^2+^ g^−1^)	Reference
Acrylamide/maleic acid hydrogels	28–81	[25]
Poly(maleic acid-alt-styrene) cross-linked with divinylbenzene resin	15.4	[26]
Poly(maleic acid-alt-styrene) cross-linked with divinylbenzene beads	17.5	[27]
Poly(maleic acid-alt-styrene) cross-linked with 1,2-diaminoethane	49.02	[58]
Poly(maleic acid-alt-styrene) modified with aminothiophene	71–100	[59]
Poly(maleic acid-alt-styrene) modified with diamines	100	[60]
CS and cross-linked CS (with epichlorohydrin)	35.5–37.9	[61]
CS and cross-linked CS (with glutaraldehyde)	61–86	[62]
Chemically modified CS	20.3	[64]
CS/silica aerogel	40	[65]
CS/zeolite composites	14.7–25.6	[63]
CS/Fe_2_O_3_/sludge biochar	55.2	[50]
Cross-linked CS (with epichlorohydrin and triphosphate)	130.7	[66]
Carboxymethyl chitosan grafted with poly(N-acryloyl glycine)	85–146	[67]
CS/starch-graft-poly(acrylonitrile) beads	85–101	[68]
CS/starch-graft-poly(amidoxyme) beads	133–233	[53]
CS modified with L-arginine/magnetic nanoparticles	142.8	[69]
CS/magnetic nanoparticles modified with L-arginine	172.4	[70]
Carboxymethylated CS beads	130	[71]
CS/malic acid beads	183.8	[72]
CS-*g*-poly(acrylic acid)	232.6	[49]
CS-*g*-maleic acid	305.5	[18]
Xanthate-modified CS/poly(acrylic acid) hydrogel	241	[16]
CS-graft-poly(acrylic acid)/biochar composite	111	[15]
CS/MA-VA3 beads	142.4	This work

**Table 5:** Thermodynamic parameters for Cu^2+^ sorption on CS/MA-VA beads.

Sorbent	Δ*H* (KJ mol^−1^)	Δ*S* (KJ mol^−1^)	Δ*G* (KJ mol^−1^)		
			298 K	308 K	318 K
CS/MA-VA1	11.0	0.127	−1.14	−1.42	−1.96
CS/MA-VA3	35.7	0.040	−2.23	−3.32	−4.78

## Data Availability

The data presented in this study are available upon request from the corresponding authors.

## References

[1] Liu Y., Wang H., Cui Y., Chen N. (2023). Removal of Copper Ions from Wastewater: A Review. Int. J. Environ. Res. Public Health.

[2] Rehman M., Liu L., Wang Q., Saleem M.H., Bashir S., Ullah S., Peng D. (2019). Copper environmental toxicology, recent advances, and future outlook: A review. Environ. Sci. Pollut. Res..

[3] Zhang L., Zeng Y., Cheng Z. (2016). Removal of heavy metal ions using chitosan and modified chitosan: A review. J. Mol. Liq..

[4] Hu Q., Lan R., He L., Liu H., Pei X. (2023). A critical review of adsorption isotherm models for aqueous contaminants: Curve characteristics, site energy distribution and common controversies. J. Environ. Manag..

[5] Darban Z., Shahabuddin S., Gaur R., Ahmad I., Sridewi N. (2022). Hydrogel-based adsorbent material for the effective removal of heavy metals from wastewater: A comprehensive review. Gels.

[6] Dragan E.S., Dinu M.V. (2020). Advances in porous chitosan-based composite hydrogels: Synthesis and applications. React. Funct. Polym..

[7] Liu C., Liu H., Zheng Y., Luo J., Lu C., He Y., Pang X. (2023). Schiff base crosslinked graphene/oxidized nanofibrillated cellulose/chitosan foam: An efficient strategy for selective removal of anionic dyes. Int. J. Biol. Macromol..

[8] Balakrishnan A., Appunni S., Chinthala M., Jacob M.M., Vo D.V.N., Reddy S.S., Kunnel E.S. (2023). Chitosan-based beads as sustainable adsorbents for wastewater remediation: A review. Environ. Chem. Lett..

[9] Guibal E. (2004). Interactions of metal ions with chitosan-based sorbents: A review. Sep. Purif. Technol..

[10] Chelu M., Musuc A.M., Popa M., Calderon Moreno J.M. (2023). Chitosan hydrogels for water purification applications. Gels.

[11] Varma A.J., Deshpande S.V., Kennedy J.F. (2004). Metal complexation by chitosan and its derivatives: A review. Carbohydr. Polym..

[12] Boamah P.O., Huang Y., Hua M., Zhang Q., Wu J., Onumah J., Sam-Amoah L.K., Boamah P.O. (2015). Sorption of heavy metal ions onto carboxylate chitosan derivatives—A mini-review. Ecotoxicol. Environ. Saf..

[13] He J., Sun F., Han F., Gu J., Ou M., Xu W., Xu X. (2018). Preparation of a novel polyacrylic acid and chitosan interpenetrating network hydrogel for removal of U(vi) from aqueous solutions. RSC Adv..

[14] Kumararaja P., Manjaiah K.M., Datta S.C., Ahammed Shabeer T.P., Sarkar B. (2018). Chitosan-g-poly(acrylic acid)-bentonite composite: A potential immobilizing agent of heavy metals in soil. Cellulose.

[15] Zhang L., Tang S., He F., Liu Y., Mao W., Guan Y. (2019). Highly efficient and selective capture of heavy metals by poly(acrylic acid) grafted chitosan and biochar composite for wastewater treatment. Chem. Eng. J..

[16] Dong L., Shan C., Liu Y., Sun H., Yao B., Gong G., Jin X., Wang S. (2022). Characterization and mechanistic study of heavy metal adsorption by facile synthesized magnetic xanthate-modified chitosan/polyacrylic acid hydrogels. Int. J. Environ. Res. Public Health.

[17] Yu Z., Dang Q., Liu C., Cha D., Zhang H., Zhu W., Zhang Q., Fan B. (2017). Preparation and characterization of poly(maleic acid)-grafted cross-linked chitosan microspheres for Cd(II) adsorption. Carbohydr. Polym..

[18] Ibrahim A.G., Saleh A.S., Elsharma E.M., Metwally E., Siyam T. (2019). Chitosan-g-maleic acid for effective removal of copper and nickel ions from their solutions. Int. J. Biol. Macromol..

[19] Chiţanu G.C., Carpov A. (2002). Ecologically benign polymers: The case of maleic polyelectrolytes. Environ. Sci. Technol..

[20] Popescu I., Thakur V.K., Thakur M.K. (2015). Pharmaceutical applications of maleic anhydride/acid copolymers. Handbook of Polymers for Pharmaceutical Technologies.

[21] Popescu I., Suflet D.M., Pelin I.M., Popa M.I. (2012). Influence of the comonomer on the dissociation of some alternating maleic acid copolymers. J. Macromol. Sci. B.

[22] Paoletti S., Delben F. (1975). A spectroscopic investigation of complexes of divalent metal ions with maleic acid copolymers. Eur. Polym. J..

[23] Mazi H., Gulpinar A. (2014). Cu(II), Zn(II) andMn(II) complexes of poly(methyl vinyl ether-alt-maleic anhydride, Synthesis, characterization and thermodynamic parameters. J. Chem. Sci..

[24] Amjad Z., Koutsoukos P.G. (2014). Evaluation of maleic acid based polymers as scale inhibitors and dispersants for industrial water applications. Desalination.

[25] Saraydin D., Karadağ E., Güven O. (1995). Adsorptions of some heavy metal ions in aqueous solutions by acrylamide/maleic acid hydrogels. Sep. Sci. Technol..

[26] Roy P.K., Rawat A.S., Choudhary V., Rai P.K. (2004). Synthesis and analytical application of a chelating resin based on a crosslinked styrene/maleic acid copolymer for the extraction of trace-metal ions. J. Appl. Polym. Sci..

[27] Gonte R., Balasubramanian K. (2016). Heavy and toxic metal uptake by mesoporous hypercrosslinked SMA beads: Isotherms and kinetics. J. Saudi Chem. Soc..

[28] Popescu I., Suflet D.M., Pelin I.M., Trunfio-Sfarghiu A.M. (2018). Crosslinked maleic anhydride copolymers microspheres for dye adsorption. Rev. Roum. Chim..

[29] Popescu I., Popa M.I., Chiţanu G.C. (2008). Supramolecular systems from natural polymers and maleic polyelectrolytes. Macromol. Symp..

[30] Krayukhina M.A., Samoilova N.A., Erofeev A.S., Yamskov I.A. (2010). Complexation of chitosan with maleic acid copolymers. Polym. Sci. Ser. A.

[31] Saleh A.S., Ibrahim A.G., Elsharma E.M., Metwally E., Siyam T. (2018). Radiation grafting of acrylamide and maleic acid on chitosan and effective application for removal of Co(II) from aqueous solutions. Radiat. Phys. Chem..

[32] Abdelmonem I.M., Elsharma E.M., Emara A.M. (2023). Radiation synthesis of chitosan/poly(acrylamide-co-maleic acid) hydrogel for the removal of 152+154Eu (III) ions. Appl. Radiat. Isot..

[33] Wei L., Chen Y., Tan W., Li Q., Gu G., Dong F., Guo Z. (2018). Synthesis, characterization, and antifungal activity of pyridine-based triple quaternized chitosan derivatives. Molecules.

[34] Sethi S., Thakur S., Sharma D., Singh G., Sharma N., Kaith B.S., Khullar S. (2022). Malic acid cross-linked chitosan based hydrogel for highly effective removal of chromium (VI) ions from aqueous environment. React. Funct. Polym..

[35] Miao W., Cheng W., Song W. (2023). The influence of poly(maleic anhydride-co-vinyl acetate) on polylactide/wood flour/calcium carbonate composites. Polym. Test..

[36] Luna C.B., da Silva Barbosa Ferreira E., Siqueira D.D., dos Santos Filho E.A., Araújo E.M. (2022). Additivation of the ethylene–vinyl acetate copolymer (EVA) with maleic anhydride (MA) and dicumyl peroxide (DCP): The impact of styrene monomer on cross-linking and functionalization. Polym. Bull..

[37] Liu S., Zhang H., Ahlfeld T., Kilian D., Liu Y., Gelinsky M., Hu Q. (2023). Evaluation of different crosslinking methods in altering the properties of extrusion-printed chitosan-based multi-material hydrogel composites. Bio-Des. Manuf..

[38] Dinu M.V., Humelnicu I., Ghiorghita C.A., Humelnicu D. (2022). Aminopolycarboxylic acids-functionalized chitosan-based composite cryogels as valuable heavy metal ions sorbents: Fixed-bed column studies and theoretical analysis. Gels.

[39] Bashir S., Hina M., Iqbal J., Rajpar A.H., Mujtaba M.A., Alghamdi N.A., Wageh S., Ramesh K., Ramesh S. (2020). Fundamental concepts of hydrogels: Synthesis, properties, and their applications. Polymers.

[40] Kessler M., Yuan T., Kolinski J.M., Amstad E. (2023). Influence of the degree of swelling on the stiffness and toughness of microgel-reinforced hydrogels. Macromol. Rapid Commun..

[41] Domard A. (1987). pH and c.d. measurements on a fully deacetylated chitosan: Application to CuII-polymer interactions. Int. J. Biol. Macromol..

[42] Zhang F., Wang M., Zhou L., Ma X., Zhou Y. (2014). Removal of Cd(II) from aqueous solution using crosslinked chitosan–zeolite composite. Desalin. Water Treat..

[43] Dinu M.V., Dragan E.S. (2010). Evaluation of Cu^2+^, Co^2+^ and Ni^2+^ ions removal from aqueous solution using a novel chitosan/clinoptilolite composite: Kinetics and isotherms. Chem. Eng. J..

[44] Yu J., Zheng J., Lu Q., Yang S., Zhang X., Wang X., Yang W. (2016). Selective adsorption and reusability behavior for Pb^2+^ and Cd^2+^ on chitosan/poly(ethylene glycol)/poly(acrylic acid) adsorbent prepared by glow-discharge electrolysis plasma. Colloid. Polym. Sci..

[45] Hernández R.B., Yola O.R., Mercê A.L.R. (2007). Chemical equilibrium in the complexation of first transition series divalent cations Cu^2+^, Mn^2+^ and Zn^2+^ with chitosan. J. Braz. Chem. Soc..

[46] Rhazi M., Desbrieres J., Tolaimate A., Rinaudo M., Vottero P., Alagui A. (2002). Contribution to the study of the complexation of copper by chitosan and oligomers. Polymer.

[47] Wang J., Guo X. (2020). Adsorption kinetic models: Physical meanings, applications, and solving methods. J. Hazard. Mater..

[48] Weber W.J., Morris J.C. (1963). Kinetics of adsorption on carbon from solution. J. Sanit. Eng. Div..

[49] Meetam P., Phonlakan K., Nijpanich S., Budsombat S. (2024). Chitosan-grafted hydrogels for heavy metal ion adsorption and catalytic reduction of nitroaromatic pollutants and dyes. Int. J. Biol. Macromol..

[50] Zhang M., Liu Y., Yin Z., Feng D., Lv H. (2023). Preparation and adsorption properties of magnetic chitosan/sludge biochar composites for removal of Cu^2+^ ions. Sci. Rep..

[51] Shaaban A.F., Fadel D.A., Mahmoud A.A., Elkomy M.A., Elbahy S.M. (2014). Synthesis of a new chelating resin bearing amidoxime group for adsorption of Cu(II), Ni(II) and Pb(II) by batch and fixed-bed column methods. J. Environ. Chem. Eng..

[52] Dahri M.K., Kooh M.R.R., Lim L.B.L. (2015). Application of Casuarina equisetifolia needle for the removal of methylene blue and malachite green dyes from aqueous solution. Alex. Eng. J..

[53] Dragan E.S., Apopei Loghin D.F., Cocarta A.I. (2014). Efficient sorption of Cu^2+^ by composite chelating sorbents based on potato starch-graft-polyamidoxime embedded in chitosan beads. ACS Appl. Mater. Interfaces.

[54] Campos N.F., Barbosa C.M.B.M., Rodríguez-Díaz J.M., Duarte M.M.B. (2018). Removal of naphthalenic acids using activated charcoal: Kinetic and equilibrium studies. Adsorpt. Sci. Technol..

[55] Allen S.J., Mckay G., Khader K.Y.H. (1989). Intraparticle diffusion of a basic dye during adsorption onto Sphagnum peat. Environ. Pollut..

[56] Srivastava V.C., Swamy M.M., Mall I.D., Prasad B., Mishra I.M. (2006). Adsorptive removal of phenol by bagasse fly ash and activated carbon: Equilibrium, kinetics and thermodynamics. Colloids Surf. A Physicochem. Eng. Asp..

[57] Hinz C. (2001). Description of sorption data with isotherm equations. Geoderma.

[58] Samadi N., Hasanzadeh R., Rasad M. (2015). Adsorption isotherms, kinetic, and desorption studies on removal of toxic metal ions from aqueous solutions by polymeric adsorbent. J. Appl. Polym. Sci..

[59] Ali E.A., Elkholy S.S., Morsi R.E., Elsabe M.Z. (2016). Studies on adsorption behavior of Cu (II) and Cd (II) onto aminothiophene derivatives of styrene maleic anhydride copolymer. J. Taiwan Inst. Chem. Eng..

[60] Abo-Baker E., Elkholy S.S., Elsabee M.Z. (2015). Modified poly (styrene maleic anhydride) copolymer for the removal of toxic metal cations from aqueous solutions. Am. J. Polym. Sci..

[61] Chen A.H., Liu S.C., Chen C.Y., Chen C.Y. (2008). Comparative adsorption of Cu(II), Zn(II), and Pb(II) ions in aqueous solution on the crosslinked chitosan with epichlorohydrin. J. Hazard. Mater..

[62] Machado M.O., Lopes E.C.N., Sousa K.S., Airoldi C. (2009). The effectiveness of the protected amino group on crosslinked chitosans for copper removal and the thermodynamics of interaction at the solid/liquid interface. Carbohydr. Polym..

[63] Wan Ngah W.S., Teong L.C., Toh R.H., Hanafiah M.A.K.M. (2013). Comparative study on adsorption and desorption of Cu(II) ions by three types of chitosan–zeolite composites. Chem. Eng. J..

[64] Rahman A. (2024). Promising and environmentally friendly removal of copper, zinc, cadmium, and lead from wastewater using modified shrimp-based chitosan. Water.

[65] Vareda J.P., Matias P.M.C., Paixão J.A., Murtinho D., Valente A.J.M., Durães L. (2024). Chitosan–silica composite aerogel for the adsorption of cupric ions. Gels.

[66] Laus R., de Fávere V.T. (2011). Competitive adsorption of Cu(II) and Cd(II) ions by chitosan crosslinked with epichlorohydrin–triphosphate. Biores. Technol..

[67] El-Sherbiny I.M. (2009). Synthesis, characterization and metal uptake capacity of a new carboxymethyl chitosan derivative. Eur. Polym. J..

[68] Dragan E.S., Apopei Loghin D.F. (2018). Fabrication and characterization of composite cryobeads based on chitosan and starches-g-PAN as efficient and reusable biosorbents for removal of Cu^2+^, Ni^2+^, and Co^2+^ ions. Int. J. Biol. Macromol..

[69] Wu Z.C., Wang Z.Z., Liu J., Yin J.H., Kuang S.P. (2016). Removal of Cu(II) ions from aqueous water by L-arginine modifying magnetic chitosan. Coll. Surf. A Physicochem. Eng. Asp..

[70] Verma R., Asthana A., Singh A.K., Prasad S. (2017). An arginine functionalized magnetic nano-sorbent for simultaneous removal of three metal ions from water samples. RSC Adv..

[71] Yan H., Dai J., Yang Z., Cheng R. (2011). Enhanced and Selective dsorption of Copper(II) Ions on Surface Carboxymethylated Chitosan Hydrogel Beads. Chem. Eng. J..

[72] Zhang Y., Lin S., Qiao J., Kołodyńska D., Ju Y., Zhang M., Cai M., Deng D., Dionysiou D.D. (2018). Malic acid-enhanced chitosan hydrogel beads (mCHBs) for the removal of Cr(VI) and Cu(II) from aqueous solution. Chem. Eng. J..

[73] Wang X., Zheng Y., Wang A. (2009). Fast removal of copper ions from aqueous solution by chitosan-g-poly(acrylic acid)/attapulgite composites. J. Hazard. Mater..

[74] Qi X., Wang Z., Ma S., Wu L., Yang S., Xu J. (2014). Complexation behavior of poly(acrylic acid) and lanthanide ions. Polymer.

[75] Gamzazade A.I., Slimak V.M., Skljar A.M., Stykova E.V., Pavlova S.S.A., Rogozin S.V. (1985). Investigation of the hydrodynamic properties of chitosan solutions. Acta Polym..

[76] Chiţanu G.C., Popescu I., Carpov A. (2006). Synthesis and characterization of maleic anhydride copolymers and their derivatives. 2. New data on the copolymerization of maleic anhydride with vinyl acetate. Rev. Roum. Chim..

[77] Aida H., Yoshida T., Matsuyama A. (1968). Dilute solution properties of maleic anhydride-vinyl acetate copolymer. Fukui Daigaku Kogakubu Kenkyo Hokoku.

[78] Leane M.M., Nankervis R., Smith A., Illum L. (2004). Use of ninhydrin assay to measure the release of chitosan from oral solid dosage forms. Int. J. Pharm..

[79] Prochazkova S., Vårum K.M., Østgaard K. (1999). Quantitative determination of chitosans by ninhydrin. Carbohydr. Polym..

[80] Shao H., Ding Y., Hong X., Liu Y. (2018). Ultra-facile and rapid colorimetric detection of Cu^2+^ with branched polyethylenimine in 100% aqueous solution. Analyst.

